# Emerging Contaminants in the Effluent of Wastewater Should Be Regulated: Which and to What Extent?

**DOI:** 10.3390/toxics12050309

**Published:** 2024-04-24

**Authors:** Weiwei Yang, Qingwei Bu, Qianhui Shi, Ruiqing Zhao, Haitao Huang, Lei Yang, Jianfeng Tang, Yuning Ma

**Affiliations:** 1 School of Chemical & Environmental Engineering, China University of Mining & Technology-Beijing, Beijing 100083, China2110340107@student.cumtb.edu.cn (Q.S.);; 2 State Key Laboratory of Urban and Regional Ecology, Research Center for Eco-Environmental Sciences, Chinese Academy of Sciences, Beijing 100085, China; 3 Key Laboratory of Urban Environment and Health, Institute of Urban Environment, Chinese Academy of Sciences, Xiamen 361021, China; 4 College of Environmental and Resource Sciences, Zhejiang University, Hangzhou 310058, China

**Keywords:** China’s WWTPs, emerging contaminants, risk, SSD, water quality criteria

## Abstract

Effluent discharged from urban wastewater treatment plants (WWTPs) is a major source of emerging contaminants (ECs) requiring effective regulation. To this end, we collected discharge datasets of pharmaceuticals (PHACs) and endocrine-disrupting chemicals (EDCs), representing two primary categories of ECs, from Chinese WWTP effluent from 2012 to 2022 to establish an exposure database. Moreover, high-risk ECs’ long-term water quality criteria (LWQC) were derived using the species sensitivity distribution (SSD) method. A total of 140 ECs (124 PHACs and 16 EDCs) were identified, with concentrations ranging from N.D. (not detected) to 706 μg/L. Most data were concentrated in coastal regions and Gansu, with high ecological risk observed in Gansu, Hebei, Shandong, Guangdong, and Hong Kong. Using the assessment factor (AF) method, 18 high-risk ECs requiring regulation were identified. However, only three of them, namely carbamazepine, ibuprofen, and bisphenol-A, met the derivation requirements of the SSD method. The LWQC for these three ECs were determined as 96.4, 1010, and 288 ng/L, respectively. Exposure data for carbamazepine and bisphenol-A surpassed their derived LWQC, indicating a need for heightened attention to these contaminants. This study elucidates the occurrence and risks of ECs in Chinese WWTPs and provides theoretical and data foundations for EC management in urban sewage facilities.

## 1. Introduction

Emerging contaminants (ECs) encompass newly discovered or recognized pollutants that pose risks to ecological environments and human health yet lack effective regulatory measures [1]. They mainly consist of PHACs, EDCs, persistent organic pollutants, and microplastics [2,3], among others. Advances in science and monitoring technology have led to increased detection of ECs in aquatic ecosystems such as rivers, lakes, and groundwater [4]. Due to the limited treatment performance of traditional water treatment processes, urban sewage still contains high concentrations of ECs even after treatment, making urban wastewater discharge a significant source of ECs [5,6,7,8]. Continuous discharge of ECs may accumulate in aquatic environments, endangering ecosystems [8]. Moreover, utilizing treated wastewater for landscaping [9], agricultural irrigation [10], and industrial reuse [11] may expose humans to ECs, thereby posing potential health risks [12,13]. Consequently, controlling EC discharge from WWTP effluent is crucial for aquatic ecosystems and human health. While governments prioritize identifying and regulating high-risk ECs [4], specific regulatory guidance is lacking. Variation in EC detection, discharge, risk, and regional characteristics necessitates determining which ECs to regulate and to what extent in WWTP effluent discharge.

Despite numerous reports on EC occurrence and risks in WWTP effluent, most focus on limited ECs in small-scale areas, leaving nationwide or larger-scale data gaps. Deep mining of nationwide data is crucial for identifying high-risk areas and EC species, informing regulation in WWTP effluent discharge. Water quality criteria (WQC) guide water quality assessment and pollution prevention. Generally, WQC derivation methods include statistical extrapolation [14], the AF method [15], and the biotic ligand model method [16]. The AF method, while simple, is subjective, leading to conservative outcomes, primarily used for risk assessment. The biotic ligand model’s incomplete theory hampers accurate baseline value derivation. In contrast, the statistical extrapolation method, with its rigorous logic and ample data, yields more objective and accurate WQC. Specifically, the statistical extrapolation method can be subdivided into species sensitivity distribution (SSD) and toxicity percentile rank (TPR) methods [14]. The SSD method, based on species sensitivity distribution theory, establishes a probability distribution model based on dose–response relationships, representing the sensitivity differences among different species and deducing the hazardous concentration for a given percentage of species, thereby protecting the ecosystem [14]. The TPR method, recommended by the US Environmental Protection Agency (US EPA) [17], considers water quality characteristics and the bioaccumulation effect of organisms but only utilizes the mean toxicity values of the four most sensitive genera. When deriving the WQC for pollutants unrelated to water quality characteristics and bioaccumulation effects, the SSD method is more capable of reflecting the overall ecological toxicity of chemicals, especially when considering the differences in species sensitivity. Therefore, WQC derived based on the SSD method hold promise in providing effective solutions for establishing discharge limits for high-risk ECs in WWTP effluent.

In this study, PHACs and EDCs were chosen as the focus, aiming to identify EC species, concentrations, risks, and high-risk areas in WWTP effluent across China, using nationwide data. High-risk ECs needing specific attention are selected, with SSD employed to calculate discharge limits. The objectives of this study include the following: (1) analyze PHACs and EDCs reported from Chinese WWTP effluent (2012–2022) to identify ECs needing attention; (2) screen high-risk ECs using the AF method; and (3) derive WQC for ECs in WWTP effluent using the SSD method.

## 2. Methodology

### 2.1. Methods of Data Collection

A comprehensive search was conducted utilizing the ISI Web of Science (https://webofscience.clarivate.cn, accessed on 30 December 2022) and China National Knowledge Infrastructure (CNKI) (https://kns.cnki.net/, accessed on 30 December 2022) to collect the relevant literature between 2012 and 2022 concerning PHACs and EDCs in WWTP effluent. The collected data focused solely on urban domestic wastewater, excluding pharmaceutical and industrial WWTPs, among others. This approach aimed to create provincial-level discharge maps specifically for PHACs and EDCs. Publications lacking clear location information were excluded from the analysis. Additionally, data from different wastewater treatment plants within the same study and data from the same wastewater treatment plant across different seasons were documented separately.

Furthermore, toxicological data on PHACs and EDCs were sourced from the ECOTOX database (https://cfpub.epa.gov/ecotox/) of the US EPA, accessed on 15 June 2023.

### 2.2. Risk Assessment Methods

To assess the ecological risk of a specific pollutant to aquatic organisms, the risk quotient (RQ) is computed by dividing the reported measured environmental concentration (MEC) by the predicted no-effect concentration (PNEC) [18]. PNEC is derived by dividing the most sensitive biological toxicity value by an AF. The selection criteria for assessment factors in the AF method are outlined in Table S4, while the PNEC values for PHACs and EDCs are provided in Tables S5 and S6. To accurately evaluate the exposure risk of regional ECs, values such as “N.D.” (not detected), “<LOD” (below the limit of detection), and “<LOQ” (below the limit of quantitation) are considered as zero in the calculation. The risk exceeding rate (RER) is then calculated by determining the ratio of the number of RQs with values greater than 0.1 to the total number of RQs.

### 2.3. Water Quality Criteria Derivation Method

The derivation of WQC using the SSD method was conducted according to the procedures outlined in the Technical Guidelines for Deriving Water Quality Criteria for Freshwater Organisms published by the Ministry of Ecology and Environment of the People’s Republic of China [19]. Initially, the SSD method was applied following the minimum data requirements specified in the freshwater biological water quality criteria derivation method. These requirements include covering at least three different trophic levels, including producers, and encompassing at least 10 species representing diverse biological groups. These groups include one species each of cyprinid fish, non-cyprinid fish, zooplankton, benthic invertebrates (e.g., mollusks, benthic crustaceans), amphibians, or other aquatic organisms belonging to different phyla from the aforementioned animals, and one species of phytoplankton or aquatic vascular plant.

For acute toxicity data, animals require exposure times of approximately 24 h for rotifers, 48 h for daphnids and midges, and 96 h for other species, while plants need an exposure time of approximately 96 h. For chronic toxicity data, animals need exposure durations of 48 h or longer for nematodes, 21 days or longer for other species, or exposure encompassing a sensitive life stage. Plants require exposure durations of 21 days or longer, or spanning at least one generation. Subsequently, the acute value for the same effect (AVE) and chronic value for the same effect (CVE) for all species of a certain EC were calculated following the Technical Guidelines for Deriving Water Quality Criteria for Freshwater Organisms [19].

The obtained AVE and CVE values were fitted using the National Ecological Environment Criteria Calculation Software [20], and four models (normal distribution, log-normal distribution, logistic distribution, and log-logistic distribution) were fitted to obtain the hazardous concentration for 5% of species derived from the toxicity data (HC_5_), root mean square error (RSME), and P(A-D) value. P(A-D) represents the Anderson–Darling test value, where a *p*-value > 0.05 indicates that the fit passes the A-D test and the model conforms to the theoretical distribution; RSME represents the root mean square error, with a smaller RSME indicating a higher accuracy of model fitting. The model fitting result (HC_5_) with *p* > 0.05 and the smallest RSME was selected to derive the WQC.

Short-term water quality criteria (SWQC) for aquatic organisms were calculated by dividing the hazardous concentration for 5% of species derived from the acute toxicity data (SHC_5_) by the short-term assessment factor (SAF). Long-term water quality criteria (LWQC) were derived by dividing the hazardous concentration for 5% of species derived from chronic toxicity data (LHC_5_) by the long-term assessment factor (LAF). The value of SAF or LAF was determined based on the number of data used for deriving the WQC. When the number of species included in the effective toxicity data exceeds 15, SAF or LAF is set to 2; when the number of species included in the effective toxicity data is less than or equal to 15, SAF or LAF is generally set to 3 [19].

## 3. Exposure and Risk of ECs

### 3.1. Exposure

A comprehensive dataset comprising 1178 data points related to 140 ECs was extracted from 43 available studies. Of these, 1116 data points were associated with 124 different PHACs, accounting for 94.74% of the total, and were sourced from 29 reports (Table S1). Additionally, 62 data points were related to EDCs, covering 16 EDCs, making up 5.26% of the total, and were obtained from 14 reports (Table S2). Notably, no studies reported both PHACs and EDCs simultaneously. Detailed information on both PHACs and EDCs can be found in Table S3. The geographical coverage of these data spanned 18 provinces in China, with significant concentrations observed in the Bohai Sea Rim region, the Yangtze River Delta, and the Pearl River Delta basins (Figure S1). Among the provinces, Beijing, Jiangsu, Shanghai, Fujian, Guangdong, and Hong Kong reported both PHACs and EDCs, while others reported only one type of EC. Moreover, EDC-related data points were relatively scarce in these provinces, accounting for only 1.75% to 27.59% of the total. The concentrations of the 140 ECs in WWTP effluent ranged widely, from 0 ng/L to 706 μg/L. Specifically, the mean concentrations of pharmaceuticals varied from 0 ng/L to 5.09 μg/L, while those of EDCs ranged from 0.84 ng/L to 2.45 μg/L.

**Figure 1:**
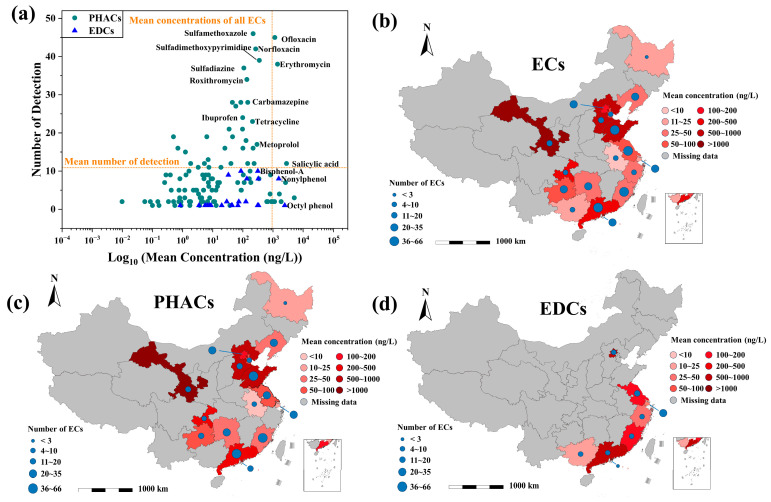
(**a**) Number of detections and mean concentration of individual ECs in the exposure database. Distribution of ECs (**b**), PHACs (**c**), and EDCs (**d**) by province recorded in the exposure.

In terms of individual ECs, sulfamethoxazole exhibited the highest detection number with 46 data points and a mean detected concentration of 219 ng/L (Figure 1a). Notably, a recent study by Guo et al. highlighted sulfamethoxazole as the most frequently detected EC in Chinese surface water, with a detection concentration of 45 ng/L [4]. Moreover, sulfamethoxazole has been detected in WWTP effluent in Germany (22.9~34.9 ng/L) [21], the United States (1640 ng/L) [22], and other countries. In addition to sulfamethoxazole, ofloxacin, erythromycin, and salicylic acid also exhibited a high detection number (exceeded the overall detection frequency, 10.8), with 45, 38, and 12 data points, respectively. Their mean detected concentrations of 1151 ng/L, 1415 ng/L, and 2819 ng/L, respectively, exceeded the mean detected concentration (934 ng/L) of all ECs. Furthermore, their mean detected concentrations generally exceeded those found in Chinese surface water [18] by tenfold, as well as being higher than those in WWTP effluent in India (ofloxacin: 0~212 ng/L; erythromycin: 1~12 ng/L) [23] and the United States (erythromycin: 230 ng/L [24]; salicylic acid: 630 ng/L [25]).

The distribution of EC exposure, in terms of composition and concentration, varied significantly among different provinces’ WWTP effluent (Figure 1b). Coastal provinces, except for Zhejiang, exhibited a relatively high number of EC exposure, with Guangdong Province having the highest number of exposed ECs at 66, followed by Fujian with 53 ECs, while Heilongjiang reported only one PHAC, caffeine. This variation could be attributed to the extensive production and consumption of chemicals in economically advanced regions, alongside heightened scientific research driven by their developed economies [4]. Regarding EC concentration, Gansu, the Bohai Sea region, and the Pearl River Delta region were identified as the most severely polluted areas. The mean EC concentrations in Gansu, Hebei, Shandong, and Guangdong were 1116 ng/L, 816 ng/L, 519 ng/L, and 354 ng/L, respectively, all exceeding the mean concentration of all ECs (258 ng/L). The high concentration of ECs in Gansu was primarily due to the high concentrations of ofloxacin (mean concentration of 9240 ng/L) and sulfapyridine (mean concentration of 1150 ng/L) among PHACs, making Gansu the province with the most severe PHAC pollution (Figure 1c). Hebei and Shandong ranked second and third in terms of PHAC pollution, with mean PHAC concentrations of 862 ng/L and 586 ng/L, respectively. The PHACs in Hebei were mainly attributed to sulfonamide (mean concentration: sulfadimethoxypyrimidine, 552.5 ng/L; sulfamethoxazole, 777.5 ng/L; sulfapyridine, 442.5 ng/L; sulfadimethoxine, 745 ng/L; sulfamerazine, 1200 ng/L; sulfathiazole, 797.5 ng/L) and beta-lactam antibiotics (mean concentration: cefazolin, 797.5 ng/L; procaine hydrochloride, 1010 ng/L; cefotaxime, 672.5 ng/L; ceftriaxone, 1867.5 ng/L; cefaclor, 890 ng/L), while in Shandong, in addition to sulfonamide (mean concentration: sulfadimethoxypyrimidine, 3967.76 ng/L; sulfamethoxypyridazine, 5086.95 ng/L; sulfamethoxazole, 267.07 ng/L) and quinolone antibiotics (mean concentration: ciprofloxacin, 390.05; enoxacin, 3264.5 ng/L; sarafloxacin, 1597.2 ng/L; oxilinic acid, 622.75 ng/L), there were also non-antibiotic PHACs such as diclofenac (mean concentration of 568 ng/L), ibuprofen (mean concentration of 525 ng/L), and carbamazepine (mean concentration of 1117 ng/L). Research on EDCs was relatively scarce, mainly concentrated in the Yangtze River Delta and the Pearl River Delta regions (Figure 1d). Hong Kong had the most severe EDC pollution, with a mean concentration of 1043 ng/L, mainly composed of nonylphenol (mean concentration of 1546 ng/L) and bisphenol A (mean concentration of 540 ng/L), followed by Guangdong Province, where major EDC species included nonylphenol (mean concentration of 2512 ng/L) and bisphenol A (mean concentration of 485 ng/L), as well as octylphenol (mean concentration of 2454 ng/L).

### 3.2. Risk

**Figure 2:**
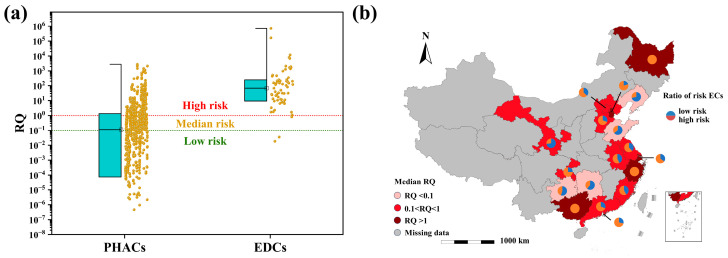
(**a**) Distribution of risk of PHACs and EDCs. (**b**) Distribution of risk by province, which was evaluated by median RQ.

Due to the limited availability of toxicity data for ECs, RQs were calculated for only 79 PHACs and 13 EDCs. As shown in Figure 2a, there was a significant difference in the overall risk between PHACs and EDCs, with median RQ values of 0.11 and 67.9, respectively, both exceeding 0.1, indicating unacceptable ecological risks. Specifically, a total of 479 data points of PHACs had risk values exceeding 0.1, accounting for 50.8% of the total data points for PHACs with calculable risks (Figure S2a). For EDCs, only five data points had RQ values < 0.1, while the remaining 54 data points had RQ values exceeding 0.1, accounting for 91.5% of the total data points for EDCs with calculable risks (Figure S2b).

Regarding the risk for individual provinces, except for Liaoning, Shandong, Guizhou, and Hunan, the median risk values for the other provinces were all higher than 0.1, indicating unacceptable risks. Nonetheless, the aforementioned four provinces still had 28.6%, 35.7%, 45.0%, and 38.7% of data points with an RQ > 0.1, signifying unacceptable risks (Figure 2b). Shandong Province reported an RQ for carbamazepine and ibuprofen of 1117.33 and 525.33, respectively, exceeding the critical value for unacceptable risks by more than three orders of magnitude, warranting special attention. Heilongjiang had the highest median RQ value, mainly due to the small data size (only two data points of caffeine). The median RQ of Zhejiang and Guangxi was also higher than 1, ranking among the highest in the country. This was attributed to the fact that only concentrations of EDCs were reported in these two regions, and since the PNEC for EDCs is generally low, this resulted in significantly higher median RQ values compared to other provinces. Furthermore, we also calculated RQ values for all data points of ECs (Figure S2). For PHACs, the median RQ values of 19 PHACs including sulfamethoxypyridazine, trimethoprim, ofloxacin, erythromycin, roxithromycin, spiramycin, tetracycline, ibuprofen, gemfibrozil, carbamazepine, bezafibrate, metoprolol, ketoprofen, caffeine, venlafaxine, fluoxetine, diazepam, oxazepam, and acetaminophen were all greater than 1, indicating high risk levels. For EDCs, except for chloroform and diethylstilbestrol, all other ECs had median RQ values exceeding 1, placing them in the high-risk category. These ECs pose a serious threat to aquatic organisms upon discharge after wastewater treatment and require close attention.

**Figure 3:**
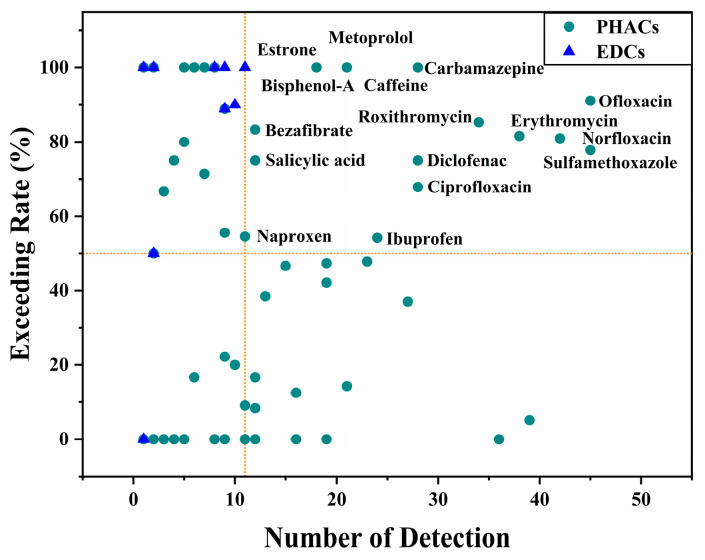
Risk exceeding rate (RER) and number of detections of ECs based on risk.

The identified high-risk ECs also exhibit a significant trend of risk exceeding rate (RER > 50%) and are detected frequently (exceeding the mean detection number). As illustrated in Figure 3, a total of 16 high-risk ECs were identified, comprising 14 PHACs and 2 EDCs. Among the PHACs are ofloxacin, norfloxacin, sulfamethoxazole, erythromycin, carbamazepine, roxithromycin, diclofenac, ciprofloxacin, caffeine, ibuprofen, metoprolol, bezafibrate, salicylic acid, and naproxen, while the EDCs include estrone and bisphenol A. The high ecological risks of these ECs have also been extensively documented in other studies (e.g., ofloxacin [26,27,28,29], norfloxacin [26,28,30,31], sulfamethoxazole [32,33,34], erythromycin [35,36,37], carbamazepine [32,38,39,40,41], roxithromycin [42,43,44,45], diclofenac [46,47,48], ciprofloxacin [49,50], caffeine [51,52,53,54], ibuprofen [38,55,56,57], metoprolol [58,59], bezafibrate [60,61], salicylic acid [62], naproxen [63,64,65], estrone [66,67,68,69], and bisphenol-A [70,71,72,73,74,75]). Moreover, these contaminants have been widely documented in surface waters across China [4,76]. Nevertheless, the current discharge standards of WWTPs in China fail to address these ECs, resulting in a lack of effective regulation over ECs with significant ecological risks.

## 4. The Derivation Water Quality Criteria of ECs

Taking into account the detection frequency, mean detection concentration, high-risk exceedance rate, and the presence of locally severe ECs in WWTP effluent in China, a total of 18 ECs including ofloxacin, norfloxacin, sulfamethoxazole, sulfapyridine, erythromycin, carbamazepine, roxithromycin, diclofenac, ciprofloxacin, caffeine, ibuprofen, metoprolol, bezafibrate, salicylic acid, bisphenol-A, estrone, nonylphenol, and octylphenol were selected for deriving WQC using the SSD method. However, only carbamazepine, ibuprofen, and bisphenol-A met the data requirements for SSD derivation. The toxicity data for these three ECs were processed according to the SSD method’s requirements for deriving short-term and long-term water quality standards for freshwater organisms. The processed toxicity data are detailed in Tables S7–S12.

**Figure 4:**
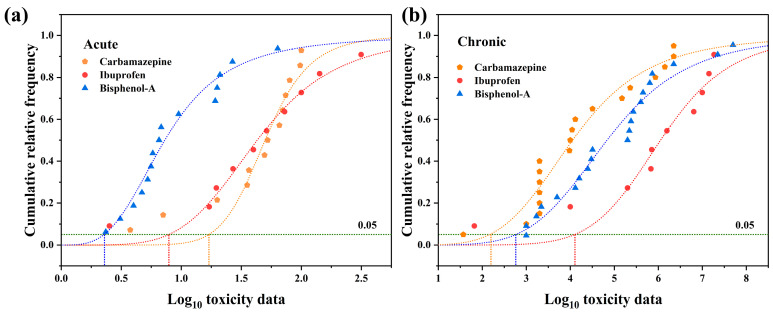
SSDs based on acute (**a**) and chronic (**b**) toxicity data for carbamazepine, ibuprofen, and bisphenol-A.

The toxicity data were inputted into the National Ecological Environment Standard Calculation Software and fitted using four models: normal distribution, log-normal distribution, logistic, and log-logistic. The resulting HC_5_, RSME, and P(A-D) values are shown in Table S13. As shown in Figure 4, the logistic model demonstrated excellent fits for the acute toxicity data of carbamazepine and ibuprofen, as well as the chronic toxicity data of ibuprofen. The log-logistic model exhibited excellent fits for the acute toxicity data of bisphenol A, the chronic toxicity data of carbamazepine, and the chronic toxicity data of bisphenol A. Further calculations revealed that the SWQC for carbamazepine, ibuprofen, and bisphenol A were 3.40 mg/L, 1.86 mg/L, and 0.89 mg/L (Table 1), respectively, which are higher than the actual concentrations of these ECs in water bodies by two to three orders of magnitude, indicating limited guidance for controlling the discharge of ECs in wastewater. However, the LWQC for carbamazepine, ibuprofen, and bisphenol A were 96.4 ng/L, 1010 ng/L, and 288 ng/L (Table 1), respectively, which are comparable to the detected concentrations of these ECs in water bodies. These LWQC can effectively reflect the exceedance of ECs in WWTP effluent. Additionally, the LWQC for carbamazepine [77] and ibuprofen [78] are consistent with those calculated by other researchers, while the LWQC for bisphenol-A [79,80] are one order of magnitude lower, which may be attributed to differences in the methodological approach employed.

**Figure 5:**
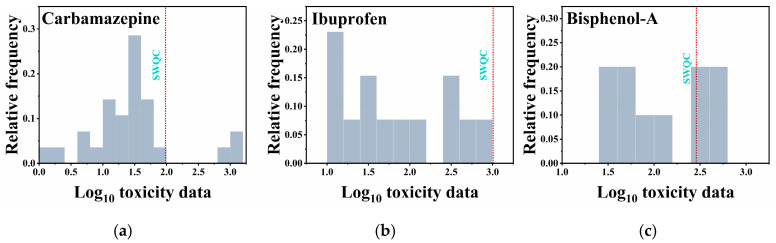
Logarithmic distribution of carbamazepine (**a**), ibuprofen (**b**), and bisphenol-A (**c**) detection data.

The feasibility of the LWQC was further validated by integrating the reported data. As depicted in Figure 5, only the reported concentration of ibuprofen did not exceed its LWQC. However, for carbamazepine and ibuprofen, 14.3% and 45.5% of the reported data points, respectively, exceeded the corresponding LWQC. Therefore, this study concludes that the derived LWQC based on the SSD method can provide valuable reference data for controlling the discharge of ECs from WWTPs in China.

## 5. Future Prospects

Exposure data analysis indicates that the volume of data reported by WWTP effluent accounts for less than one-tenth of that reported for surface water [4]. Among the 34 provincial-level administrative regions in China, only 18 provinces reported the occurrence of ECs in WWTP effluent, with some provinces having very limited data. The reported data mainly focus on PHACs, while reports on EDCs are relatively scarce. The insufficient data significantly impair the accuracy of drawing a comprehensive and precise nationwide map of ECs in WWTP effluent, making it challenging to comprehensively and accurately understand the occurrence of ECs in WWTP effluent and the corresponding risks. Furthermore, the lack of toxicity data for some ECs prevents the calculation of their risk values, further exacerbating the limited understanding of EC risk levels in WWTP effluent. Therefore, future efforts should focus on generating more data to create a more objective and comprehensive fine-scale map of the occurrence and risk of ECs in WWTP effluent nationwide.

Regarding toxicity data, among the 18 high-risk ECs identified in this study, only three meet the basic requirements for deriving WQC using the SSD method, thus making it impossible to derive scientific and objective WQC for other high-risk ECs. In fact, we further screened the toxicity data of all PHACs with unacceptable risks based on the requirements of the SSD method. It was found that even for some PHACs with higher risk exceedance rates and detection frequencies, such as sulfamethoxazole, tetracycline, and erythromycin, although the toxicity data are adequate, WQC cannot be derived based on the SSD method. Therefore, it is necessary to derive WQC for more ECs based on more comprehensive and complete toxicity data to scientifically, accurately, objectively, and effectively control the discharge risks of ECs in WWTP effluent in the future.

## 6. Conclusions

In summary, we comprehensively investigated the occurrence and risks of PHACs and EDCs in WWTP effluent in China based on exposure data spanning from 2012 to 2022. Monitoring a total of 140 emerging contaminants (ECs), including 124 PHACs and 16 EDCs, revealed concentrations ranging from 0 to 706 μg/L. PHACs dominated the dataset, constituting 94.02% of the total. Through analyses encompassing overall exposure concentrations, regional risk assessment, risk exceedance rates, and detection frequencies, 18 ECs emerged as requiring close attention. These ECs were ofloxacin, norfloxacin, sulfamethoxazole, sulfapyridine, erythromycin, carbamazepine, roxithromycin, diclofenac, ciprofloxacin, caffeine, ibuprofen, metoprolol, bezafibrate, salicylic acid, bisphenol-A, estrone, nonylphenol, and octylphenol. Utilizing the SSD method, WQC were derived for three ECs meeting the derivation requirements: carbamazepine, ibuprofen, and bisphenol A, with respective LWQC of 96.4 ng/L, 1010 ng/L, and 288 ng/L. Except for ibuprofen, exposure data for carbamazepine and bisphenol A surpassed their LWQC to varying extents, underscoring the significance of these standards in regulating EC discharge in WWTP effluent. This study identifies major high-risk ECs and establishes LWQC based on nationwide data, offering a viable strategy for managing ECs discharge in WWTP effluent.

## Figures and Tables

**Table 1:** Water quality criteria calculation results.

Chemicals	Acute				Chronic			
	Model	SHC_5_ (mg/L)	SAF	SWQC (mg/L)	Model	LHC_5_ (ng/L)	LAF	LWQC (ng/L)
Carbamazepine	Logistic distribution	10.19	3	3.40	Log-normal distribution	192.8	2	96.4
Ibuprofen	Logistic distribution	5.59	3	1.86	Logistic distribution	3039.9	3	1010
Bisphenol-A	Log-logistic distribution	2.66	3	0.89	Normal distribution	575.3	2	288

## Data Availability

Data are contained within the article and Supplementary Materials.

## Supplementary Materials

The following supporting information can be downloaded at https://www.mdpi.com/article/10.3390/toxics12050309/s1: Table S1. Concentration levels of PHACs in the effluent of WWTPs in China [81,82,83,84,85,86,87,88,89,90,91,92,93,94,95,96,97,98,99,100,101,102,103,104,105,106,107,108,109]; Table S2. Concentration levels of EDCs in the effluent of WWTPs in China [110,111,112,113,114,115,116,117,118,119,120,121,122,123]; Table S3. Database of emerging contaminants detected in the effluent of WWTPs in China; Table S4. The selection criteria for AF in the derivation of PNEC; Table S5. PNEC of PHACs [124,125,126,127,128,129,130,131,132,133,134,135,136,137,138,139,140,141,142,143,144,145,146,147,148,149,150,151,152,153,154,155,156,157,158,159,160,161,162,163,164,165,166,167,168,169,170,171,172,173,174,175,176,177,178,179,180,181]; Table S6. PNEC of EDCs [174,182,183,184,185,186,187,188,189,190,191,192,193]; Table S7. Chronic toxicity data of carbamazepine on freshwater organisms [124,167,194,195,196,197,198,199,200,201,202,203,204]; Table S8. Acute toxicity data of carbamazepine on freshwater organisms [176,195,201,202,205,206,207,208,209,210,211]; Table S9. Chronic toxicity data of ibuprofen on freshwater organisms [167,168,212,213,214,215,216,217]; Table S10. Acute toxicity data of ibuprofen on freshwater organisms [155,176,209,211,214,217,218,219]; Table S11. Chronic toxicity data of bisphenol on freshwater organisms [191,220,221,222,223,224,225,226,227,228,229,230,231,232,233,234,235,236]; Table S12. Acute toxicity data of bisphenol on freshwater organisms [228,232,234,236,237,238,239,240,241,242,243,244]; Table S13. SSD fitting results of carbamazepine, ibuprofen, and bisphenol-A; Figure S1. Overview of the exposure database, including the ratio of number of detection ECs and the number of EC data by province; Figure S2. Boxplots for the calculated RQs for PHACs (a) and EDCs (b) detected in the effluent of WWTPs in China; Figure S3. The names and geographical locations of provinces in China.

## Abbreviations

Wastewater treatment plants, WWTPs; pharmaceuticals, PHACs; endocrine-disrupting chemicals, EDCs; long-term water quality criteria, LWQC; assessment factor, AF; species sensitivity distribution, SSD; not detected, N.D.; limit of detection, LOD; limit of quantitation, LOQ; toxicity percentile rank, TPR; US Environmental Protection Agency, US EPA; acute value for the same effect, AVE; chronic value for the same effect, CVE; China National Knowledge Infrastructure, CNKI; risk quotient, RQ; measured environmental concentration, MEC; predicted no-effect concentration, PNEC; hazardous concentration for 5% of species derived from toxicity data, HC_5_; root mean square error, RSME; Anderson–Darling test, P(A-D); short-term water quality criteria, SWQC; hazardous concentration for 5% of species derived from acute toxicity data, SHC_5_; short-term assessment factor, SAF; hazardous concentration for 5% of species derived from chronic toxicity data, LHC_5_; long-term assessment factor, LAF.
