# Supplementary material for: Emerging Contaminants in the Effluent of Wastewater Should Be Regulated: Which and to What Extent?
Source: Toxics. 2024 Apr 24;12(5):309. doi: 10.3390/toxics12050309 (PMC11125804; doi:10.3390/toxics12050309)
Supplement: Supplementary file 1 [file toxics-12-00309-s001.zip › Supplementary Materials.pdf]

Supporting Information

Tables (shown in "**SI Table.xlsx**")

Table S1 Concentration levels of PHACs in the effluent of WWTPs in China.

Table S2 Concentration levels of EDCs in the effluent of WWTPs in China.

Table S3 Database of emerging contaminants detected in the effluent of WWTPs in China.

Table S4 The selection criteria for AF in the derivation of PNEC.

Table S5 PNEC of PHACs.

Table S6 PNEC of EDCs.

Table S7 Chronic toxicity data of Carbamazepine on freshwater organisms.

Table S8 Acute toxicity data of Carbamazepine on freshwater organisms.

Table S9 Chronic toxicity data of Ibuprofen on freshwater organisms.

Table S10 Acute toxicity data of Ibuprofen on freshwater organisms.

Table S11 Chronic toxicity data of Bisphenol on freshwater organisms.

Table S12 Acute toxicity data of Bisphenol on freshwater organisms.

Table S13 SSD fitting results of carbamazepine, ibuprofen and bisphenol-A.

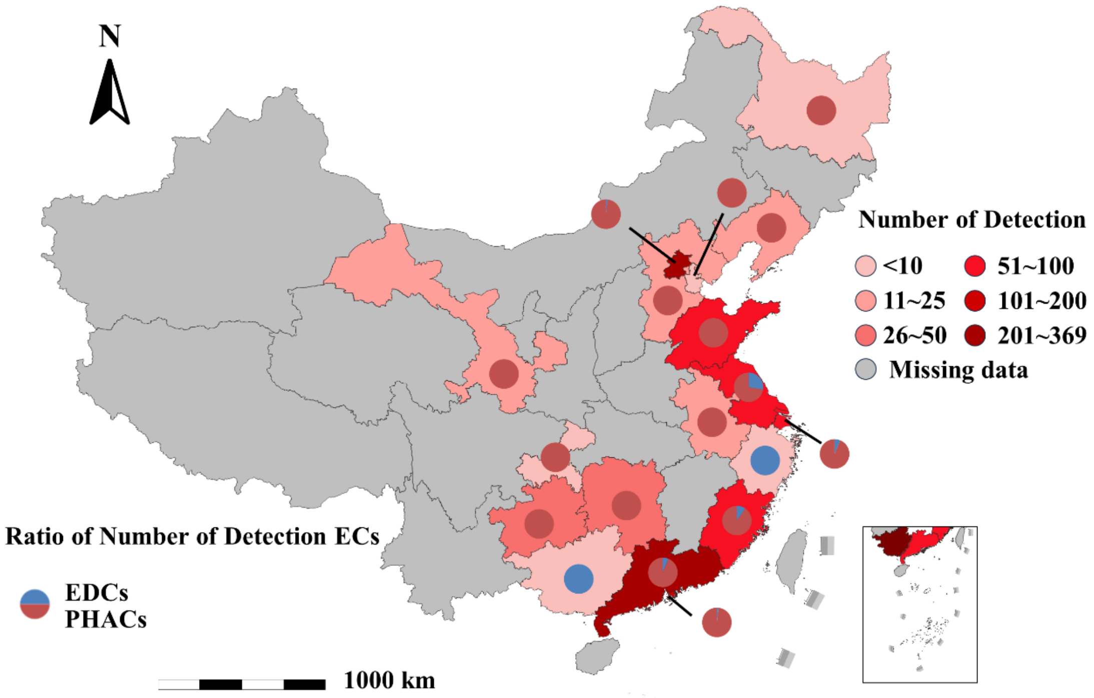

Figure S1 Overview of the exposure database, including the ratio of number of detection ECs and the number of ECs data by province.

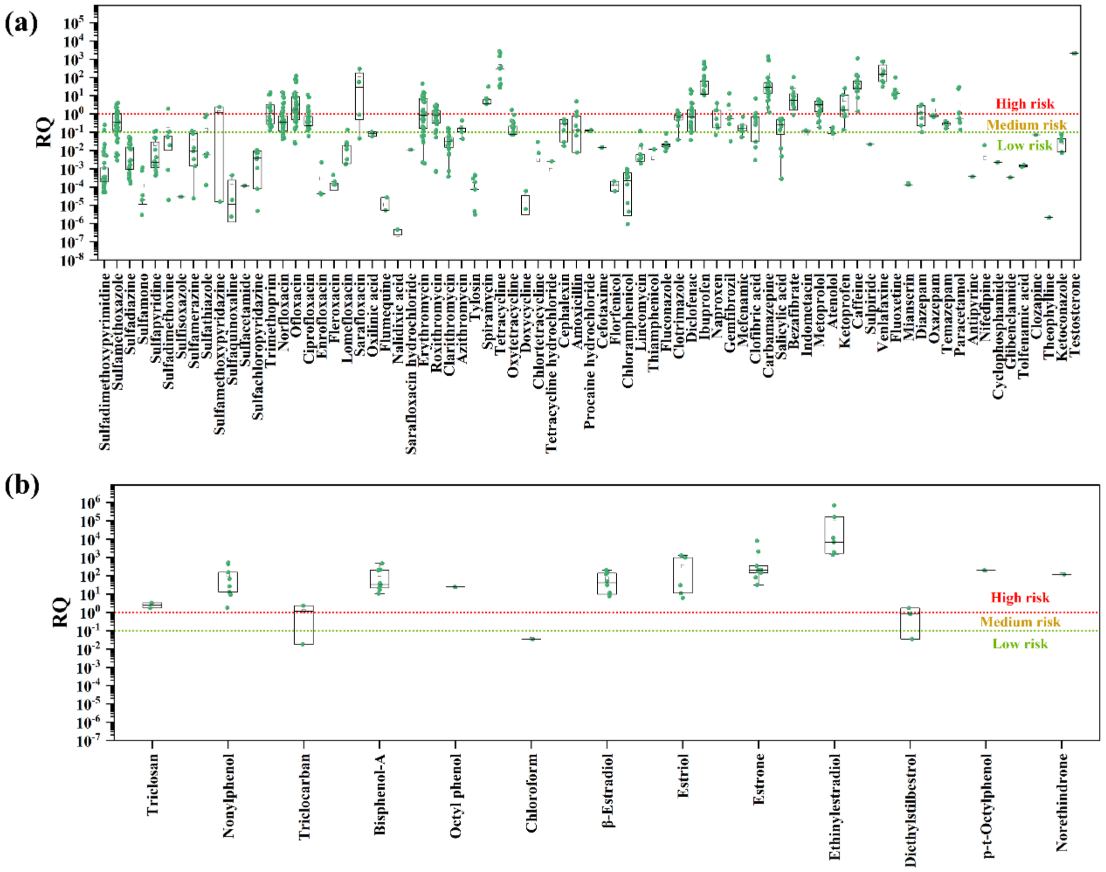

Figure S2 Boxplots for the calculated RQs for PHACs (a) and EDCs (b) detected in the

effluent of WWTPs in China.

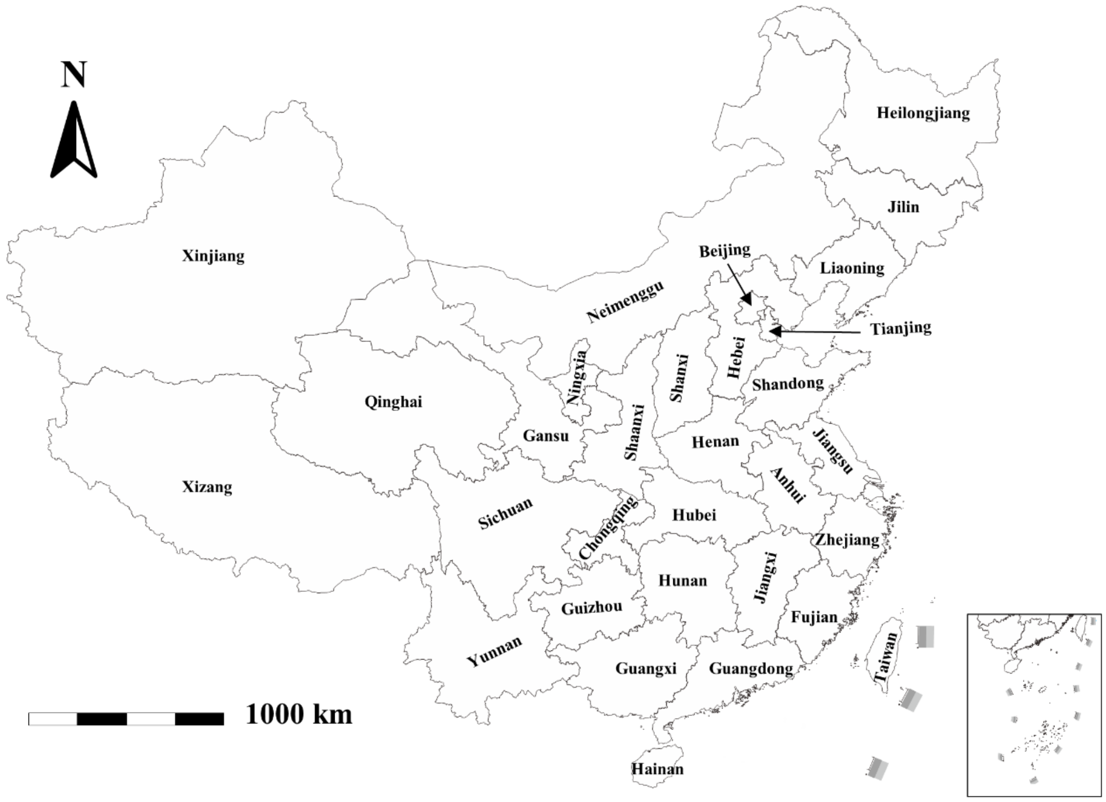

Figure S3 The names and geographical locations of provinces of China.
